# A pilot study of the Tobacco Treatment Guidelines for High-Risk Groups (TOB-G) for pregnant and postpartum women

**DOI:** 10.18332/ejm/99543

**Published:** 2018-11-28

**Authors:** Sophia Papadakis, Vergina Konstantina Vyzikidou, Victoria G. Vivilaki, Constantine I. Vardavas, Andriani N. Loukopoulou, Theodosia Peleki, Vaso Evangelopoulou, Panagiotis Behrakis

**Affiliations:** 1 George D. Behrakis Research Lab, Hellenic Cancer Society, Athens, Greece; 2 Institute of Public Health, American College of Greece, Athens, Greece; 3 Division of Prevention and Rehabilitation, University of Ottawa Heart Institute, Ottawa, Canada; 4 Faculty of Medicine, University of Ottawa, Ottawa, Canada; 5 Midwifery Department, University of West Attica, Athens, Greece; 6 Hellenic Centre for Disease Control and Prevention, Athens, Greece; 7 General Oncological Hospital of Kifisia, Athens, Greece; 8 Biomedical Research Foundation of the Athens Academy, Athens, Greece; 9 Academy of Medicine, University of Athens, Athens, Greece

**Keywords:** smoking cessation, pregnancy, tobacco, midwives, guidelines, TOB-G

## Abstract

**INTRODUCTION:**

Maternal smoking constitutes a significant risk to the fetus and is associated with multiple adverse pregnancy outcomes. Despite this, an estimated 6–19% of women in Europe smoke during pregnancy. We conducted a pilot study to examine the feasibility and effectiveness of the clinical practice recommendations of the 2017 Tobacco Cessation Guidelines for High-Risk Groups (TOB-G) for pregnant and postpartum women in an outpatient obstetrics setting.

**METHODS:**

The guideline recommendations were tested on a sample of 67 pregnant women recruited from obstetrics outpatient visits. Pregnant women who smoked received three behavioural counselling sessions through a combination of face-toface and telephone consultations by a midwife trained in the TOBG tobacco treatment recommendations. Smoking status was assessed at 1 month and at 6 months follow-up via self-report.

**RESULTS:**

Seventy-one per cent of pregnant smokers screened agreed to participate in the counselling intervention. Pregnant women participants (mean age, M=31.73 years, SD±6.09) smoked for an average of 12.2 (SD±6.55) years. Women reported smoking an average of 4.82 (SD±4.14) cigarettes per day with 51% reporting smoking within 30 minutes of waking, an indicator of higher levels of nicotine addiction. Rates of smoking abstinence among pregnant women undergoing the counselling intervention were 43.9% and 45.6% at the 1 month and at 6 months follow-up, respectively. Replacing those participants with missing data as smokers, the quit rates were 26.9% and 38.8% at the 1 month and 6 months follow-up, respectively.

**CONCLUSIONS:**

The counselling intervention delivered to pregnant women who smoke was feasible to implement in a manner that was consistent with the TOB-G guideline recommendations in an outpatient obstetrics setting. Future work should focus on increasing uptake of evidence-based tobacco treatment recommendations in outpatient obstetrics settings.

## INTRODUCTION

Smoking during pregnancy constitutes a significant risk to the fetus and is associated with multiple adverse pregnancy outcomes, including low birth weight, premature birth, and perinatal mortality that can extend into childhood^1-4^. Despite these risks, 6–19% of Europe women will smoke during pregnancy and a large portion of women who quit will return to smoking following the birth of their child^5,6^. Many of the serious adverse effects of smoking can be reversed if smoking is discontinued in the first trimester of pregnancy^7-9^. Evidence has shown that women who quit smoking during the first 3–4 months of pregnancy, give birth to infants of similar weight to those that never smoke^7,9^.

It is important that all health professionals, including family physicians, midwives, obstetricians and gynaecologist, and nurses, who work with pregnant women, be familiar with the latest evidence and be comfortable intervening and supporting women to achieving cessation^10^. Midwives are uniquely positioned to deliver education and counselling on tobacco use to pregnant women. The Tobacco Cessation Guidelines for High-Risk Groups (TOB-G) for pregnant and postpartum women were published in 2017^1^. The TOB-G guidelines provide evidence-based clinical practice recommendations for health professionals working with pregnant women who smoke. The guidelines can be accessed at http://tob-g.eu/tobacco-cessationguidelines-for-high-risk-populations-2/.

We conducted a pilot study to examine the feasibility and effectiveness of the clinical practice recommendations of the TOB-G for pregnant and postpartum women in an outpatient obstetrics setting.

## METHODS

### Design

We evaluated the feasibility and effectiveness of a smoking cessation intervention delivered by midwives trained in the TOB-G guidelines. The primary-outcome measure, a 7-day prevalence smoking abstinence, was assessed at 1 month (±2 weeks) and at 6 months (±2 weeks) for all pregnant women sampled^14^. Research Ethics approval was obtained from the General Maternity District Hospital ‘HELENA VENIZELOU’, Athens, Greece.

### Participants

A sample of pregnant women was recruited from outpatient obstetrics visits at the ‘Helena Venizelou’ General Maternity District Hospital, Athens, Greece. During the recruitment period two research assistants screened consecutive women seen in the outpatient obstetrics clinic. Eligible participants were: 1) daily tobacco users (>1 cigarettes per day), 2) interested in quitting smoking in the next 30 days, 3) currently pregnant, 4) able to complete the study survey, 5) have access to a telephone for follow-up. Eligible women willing to participate in the study provided written informed consent.

### Description of the intervention

**Figure 1:**
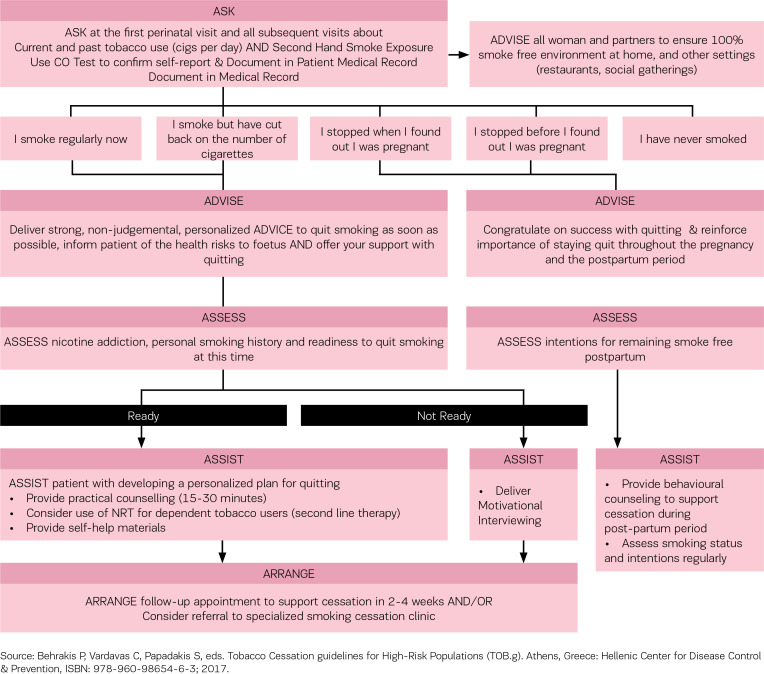
The 5As model for smoking cessation in pregnancy and the postpartum period

The intervention was based on the TOB-G recommendations and an adaptation of the 5As (ask, advise, assess, assist, arrange) model for tobacco treatment delivery in clinical settings presented in the TOB-G guidelines for pregnant women (Figure 1). While all health care professionals working with pregnant women have an important role to play in addressing tobacco use and secondhand smoke exposure, the present intervention was delivered solely by midwives. Women received an initial in-person smoking cessation counselling session from a midwife. Up to three follow-up sessions were delivered via telephone at 1 month, and at 2 and 6 months. A printed self-help resource was provided to all participants. Participating pregnant women worked with a midwife to develop a quitplan based on individual smoking and personal history. The initial counselling focussed on strategies for addressing motivation, cravings and withdrawal in preparation for their quit-date and reviewed personal triggers for tobacco use. At each of the follow-up sessions pregnant women were provided with brief counselling and discussed strategies for addressing cravings, withdrawal, and high-risk situations.

### Data collection procedures

All consenting women completed a baseline survey to document sociodemographic characteristics (age, gender) and medical and smoking history. Smoking history included: number of years of smoking, number of cigarettes per day, and time to first cigarette in the morning, with smoking within 30 minutes of waking in the morning being a proxy for higher levels of nicotine dependence^11^. The study research assistant coordinated screening, consenting and baseline data collection activities. The research assistant contacted participants by phone at 1 month and at 6 months later to document smoking status. Three phone calls were placed before categorizing non-respondents as lost to follow-up.

### Statistical analysis

Descriptive statistics were used to summarize sociodemographic characteristics. We reported rates of smoking abstinence for those participants successfully reached by phone. In a second analysis, women who were not reached for follow-up were categorized as active smokers, based on the Russell Standard for reporting smoking cessation outcomes^12^. Logistic regression was used to examine the association between participants’ age, years smoking, cigarettes smoked per day, and time to first cigarette in the morning. Statistical analysis was performed with the use of the Statistical Package for Social Sciences (SPSS), 24th edition.

## RESULTS

### Participants

A total of 94 women screened were eligible to participate and reported current tobacco use. Seventy-one per cent of pregnant smokers screened agreed to participate in the counselling intervention. A sample of 67 pregnant women was recruited. Data were available for 79.1% and 85.1% of the participating pregnant women, at the 1 month and at 6 months follow-up, respectively. Table 1 summarizes the sociodemographic characteristics of pregnant women sampled. Pregnant women (mean age, M=31.73 years, SD±6.09) smoked on average 12.2 (SD±6.55) years, with more than half reporting smoking for more than ten years.

**Table 1:** Characteristics of participating pregnant women (n=67)

*Variable*	*n (%)*
**Age (years)**	
<20	3 (4.5)
20–29	19 (28.4)
30–39	37 (55.2)
≥40	8 (12.0)
**Cigarettes/day**	
1–5	48 (71.6)
6–10	15 (22.4)
11–19	3 (4.5)
≥20	1 (1.5)
**Years of smoking**	
≤ 5	12 (17.9)
6–10	19 (28.4)
11–20	31 (46.3)
>20	5 (7.5)
**Minutes after waking to first cigarette**	
≤ 5	14 (20.9)
6–30	20 (29.9)
31–60	6 (9.0)
>60	27 (40.3)

At the time of the baseline assessment participating women reported smoking an average of 4.82 (SD±4.14) cigarettes per day with the majority (71.6%) of women reporting they smoked 5 or less cigarettes per day at baseline. Almost 51% of the women reported smoking within 30 minutes of waking, an indicator of higher levels of nicotine addiction.

### Cessation outcomes

Self-reported smoking abstinence was 43.9% and 45.6% at the 1 month and 6 months follow-up, respectively (Table 2). Replacing those participants with missing data as smokers, the quit rates were 26.9% and 38.8% at the 1 month and 6 months follow-up, respectively (Table 2). Among the predictor variables examined there were no significant associations with smoking status at 1 month and at 6 months.

**Table 2:** Proportion of pregnant women selfreporting abstinence at the 1 month and at 6 months follow-up

*Variable*	*n (%)*
**At 1 month**	
Participants reached	18/41 (43.9)
Missing data replaced as smokers	18/67 (26.9)
**At 6 months**	
Participants reached	26/57 (45.6)
Missing data replaced as smokers	26/67 (38.8)

## DISCUSSION

The counselling intervention delivered to pregnant women who smoked was feasible to implement in an outpatient obstetrics setting by midwives in a manner that was consistent with the TOB-G guideline recommendations. Among pregnant women who smoked there was high levels of uptake of the counselling intervention that increased smoking abstinence.

Based on the TOBG guideline recommendation, the intervention was delivered through a combination of face-to-face and telephone counselling and involved four contacts in total. Counselling was provided for approximately 15 minutes at each contact when possible, as this is one of the recommended practices for addressing tobacco use among pregnant women. Our results are consistent with those of other published reports and recent meta-analysis by the Cochrane review that emphasizes the need for a minimum of 15 minutes of behavioural counselling and follow-up support in order to support cessation among pregnant women^13^.

Despite the rates of cessation documented at the 6 months follow-up, a significant number of women continued to smoke. More intensive interventions are needed, including frequent face-to-face, based on the TOB-G guidelines. Given the high rates of nicotine addiction reported in the present study and as recommended by the TOBG-G guidelines, women unable to quit smoking may require use of nicotine replacement therapy as a harm reduction strategy^11^. It is known that tobacco use among women during pregnancy is influenced by a partner’s tobacco use as well as tobacco use among family members and by others in the home and immediate environment^14-17^. Addressing tobacco use among family members may further increase cessation outcomes, although at present there is limited research in this area^1,13^. Also, high levels of maternal stress and depression are associated with tobacco use during pregnancy and may require more tailored intervention than that offered in the present study^14,16,18,19^. The present study did not include screening or tailored intervention related to stress and depression or partners smoking status, which would be of value to examine in future research. Overall there is a need for more research to inform evidencebased practice in the treatment of tobacco use during pregnancy^1,20-21^.

While there is good evidence that tobacco treatment counselling is effective is supporting cessation among pregnant women who smoke, it is not currently delivered as part of maternal care in Europe. Several barriers have been previously documented among health care professionals^22,23^. The integration of evidence-based practices for addressing tobacco use among pregnant women who smoke as a standard of practice in obstetrics settings should be the focus of future work. Specifically, offering all women who smoke advise to quit smoking and structured assistance with quitting using counselling, and as appropriate quit smoking medications as part of their care from the obstetrics service or via referral to a community-based cessation service, is necessary for improving rates of cessation for women unable to quit on their own. Given the importance of quitting in the first trimester of pregnancy this should be considered a priority for the care of mother and foetus. The TOB-G guidelines recommend that midwives, as well as other health care professionals, be knowledgeable in 5As delivery and should have responsibilities for supporting cessation among women who smoke. Ensuring adequate knowledge of these evidence-based guidelines and training of midwives and other health care professionals is critical to supporting uptake of smoking cessation treatment in this high-risk population. Previous research has found low levels of knowledge and training in smoking cessation among midwives^23^. Training in best practices and counselling techniques, as such, should be integrated in undergraduate and postgraduate training.

### Limitations

The findings of our pilot study should be understood in light of the study’s methodological limitations. First, our study was conducted in a single hospital from one European city and may not be generalizable to other cities or countries. We used a pre-post design and as such we can only report on associations between exposure to the intervention and the effects documented on outcomes of interest. Additionally, self-report was used to assess smoking outcomes and may be subject to response bias. The sample size of 67 pregnant women means we had limitations in terms of identifying statistical significance in the regression analysis performed. A larger study, and possibly a randomized design, would assist with validating the findings of the present study and increasing the strength of evidence in terms of the effects of the TOB-G guidelines on cessation outcomes. Despite these limitations, this study is one of the few to examine the feasibility and uptake of European Tobacco Treatment guidelines in ‘real-world’ obstetrics settings.

## CONCLUSIONS

The counselling intervention delivered to pregnant women who smoke was feasible to implement in a manner that was consistent with the TOB-G guideline recommendations in an outpatient obstetrics setting. Future work should focus on increasing uptake of evidence-based tobacco treatment recommendations in outpatient obstetrics settings, including training health-care professionals in evidencebased practice.

## CONFLICTS OF INTEREST

S. Papadakis reports grants from Global Bridges (and funding from educational grant from Pfizer Global Inc.), outside the submitted work. In addition, V. G. Vivilaki reports that she is the Editor-in-chief of EJM journal and that there are no conflicts of interest with this current work. The rest of the authors also have completed and submitted an ICMJE form for disclosure of potential conflicts of interest. The authors declare that they have no competing interests, financial or otherwise, related to the current work.
